# Targeting Glutamine Addiction in Gliomas

**DOI:** 10.3390/cancers12020310

**Published:** 2020-01-29

**Authors:** Marta Obara-Michlewska, Monika Szeliga

**Affiliations:** Department of Neurotoxicology, Mossakowski Medical Research Centre, Polish Academy of Sciences, 5 Pawińskiego Street, 02-106 Warsaw, Poland; martomich@gmail.com

**Keywords:** glioma, glutamine, glutamate, glutaminase, glutamine synthetase, glutamate dehydrogenase, therapy

## Abstract

The most common malignant brain tumors are those of astrocytic origin, gliomas, with the most aggressive glioblastoma (WHO grade IV) among them. Despite efforts, medicine has not made progress in terms of the prognosis and life expectancy of glioma patients. Behind the malignant phenotype of gliomas lies multiple genetic mutations leading to reprogramming of their metabolism, which gives those highly proliferating cells an advantage over healthy ones. The so-called glutamine addiction is a metabolic adaptation that supplements oxidative glycolysis in order to secure neoplastic cells with nutrients and energy in unfavorable conditions of hypoxia. The present review aims at presenting the research and clinical attempts targeting the different metabolic pathways involved in glutamine metabolism in gliomas. A brief description of the biochemistry of glutamine transport, synthesis, and glutaminolysis, etc. will forego a detailed comparison of the therapeutic strategies undertaken to inhibit glutamine utilization by gliomas.

## 1. Introduction

The metabolism of neoplasms has evolved to meet the demands of their high proliferative activity and growth in adverse conditions of hypoxia, nutrient shortage, and immunological pressure of the host. The reprogrammed metabolism of neoplasms, eventually adapting them to specific growth requirements and conditions, involves addiction to glucose (the Warburg effect, oxidative glycolysis) and/or glutamine. Glucose addiction manifests as increased glucose uptake and lactate production, carried out regardless of the oxygen availability. Glycolysis, which is much less effective than oxidative phosphorylation, has developed as an adaptation to hypoxic conditions. However, high but energetically futile glycolytic rates cannot compensate for high ATP demands; therefore, neoplasms, including gliomas, also generate it via the tricarboxylic acid (TCA) cycle and oxidative phosphorylation [1,2]. Although both of those metabolic aberrations are present in gliomas [3,4,5], and are coupled [6,7], the present review focuses on glutamine addiction, and the Warburg effect will not be discussed.

## 2. A Concise Overview of Glioma Biology

Primary central nervous system (CNS) tumors are divided into over 100 histologically distinct types, each with its own spectrum of clinical presentations, treatments, and outcomes. The term glioma refers to CNS tumors that develop either from glial or precursor cells. Gliomas account for approximately 25% of all primary CNS tumors and more than 80% of malignant ones. The most common gliomas are those of astrocytic origin, and the most aggressive of them, glioblastoma (GBM, WHO grade IV), accounts for more than 57% of these neoplasms. Gliomas are characterized by a high mortality resulting mainly from the localization, high proliferative potential, infiltrative growth pattern, as well as intratumoral heterogeneity. Only 30% of patients with anaplastic astrocytoma (AA, WHO grade III) and less than 7% of patients with GBM survive five years after diagnosis [8].

Recent progress in molecular profiling has led to the identification of several genetic alterations characteristic for the particular types and malignancy grades of gliomas. This knowledge has improved the diagnostics and classification system. Briefly, somatic mutations affecting the R132 residue of the isocitrate dehydrogenase 1 (*IDH1*) or R172 residue of the isocitrate dehydrogenase 2 gene (*IDH2*) are often detected in WHO grade II or III gliomas and oligodendrogliomas. These mutations are also observed in GBM that have evolved from the lower grade gliomas (secondary GBM) but are rare in GBM patients without a clinical diagnosis of a lower grade precursor neoplasm (primary GBM). Tumors with *IDH1* or *IDH2* mutations have distinctive genetic and clinical features, and patients with such tumors have a longer overall survival time compared to patients with wild-type gliomas [9]. IDH enzymes produce α-ketoglutarate (αKG) from isocitrate, and mutations at R132 in *IDH*1 or R172 in *IDH*2 confer neomorphic enzyme activity that catalyzes the reduction of αKG into oncometabolite D-2-hydroxyglutate (D2HG). Although, intuitively, these mutations are believed to be oncogenic, their role in gliomagenesis seems to be much more complex, and the beneficial effects of mutated *IDH* enzymes compared to the wild type have been supported by several experimental and clinical findings (exhaustively reviewed in [10]).

Aside from *IDH1*/*2* mutations, two other alterations serve as diagnostic or prognostic markers. Oligodendroglial tumors often present as a 1p/19q codeletion associated with a favorable prognosis and sensitivity to chemotherapy. Approximately 40% of gliomas display methylation of the promoter region of *MGMT* coding for a DNA repair enzyme that mediates resistance to alkylating agents, such as temozolomide (TMZ). *MGMT* promoter methylation serves as both a predictive and prognostic marker in patients with GBM (reviewed in [11]).

*IDH1/2* mutation, 1p/19q codeletion, and *MGMT* promoter methylation have become integral components of brain tumor classification. The other relevant alterations that drive the pathogenesis of glioma include amplification of the gene coding for epidermal growth factor receptor (EGFR) mutations in the genes encoding telomerase reverse transcriptase (TERT) and tumor suppressor p53, as well as promoter methylation in genes coding for retinoblastoma protein (RB) and cyclin-dependent kinase inhibitor 2A (CDKN2A). Moreover, numerous other epigenetic and genetic alterations as well as deregulated gene expression lead to modifications of several signaling pathways, like the p53, RB, receptor tyrosine kinase (RTK), Ras/MAPK, phosphatidylinositol 3-kinase (PI3K)/phosphatase, and tensin homolog (PTEN)/AKT pathways (reviewed in [12]).

A growing body of evidence clearly shows that cancer stem cells (CSCs) play a crucial role in tumor relapse and metastasis. Identified for the first time in brain tumors by Singh et al., glioblastoma stem cells (GSCs) possess a capacity for proliferation, self-renewal, and differentiation [13], as well as the ability to initiate tumors in vivo [14]. Although their biology has not yet been completely unveiled, GSCs have been shown to be involved in resistance to therapies, angiogenesis, invasion, and recurrence (reviewed in [15]). The targeting of GSCs is most likely essential in order to achieve long-lasting therapeutic effects.

## 3. Glutamine in the Normal Brain

In healthy organisms, glutamine is required for the TCA cycle anaplerosis, and the synthesis of amino acids and proteins, purines/pyrimidines, nicotinamide adenine dinucleotide (NAD), and hexosamines. Additionally, glutamine also drives the uptake of essential amino acids, activates the mammalian target of rapamycin (mTOR) pathway, and its metabolism regulates pH via the NH_3_/NH_4_^+^ balance and oxidative stress through glutathione (GSH) synthesis [16,17].

**Figure 1:**
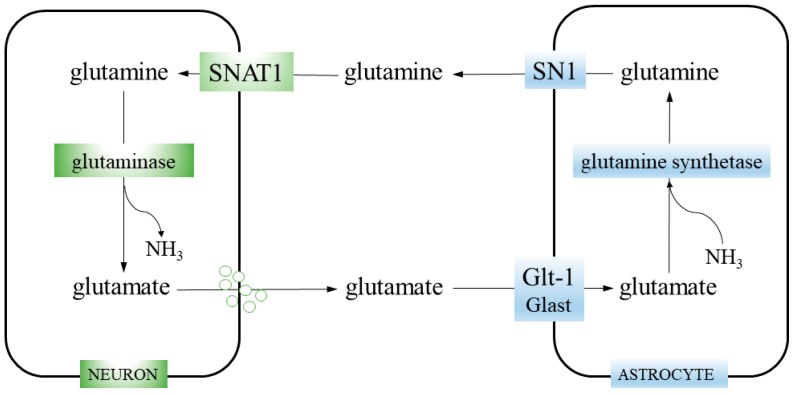
Glutamine–glutamate cycle. Neurons take up glutamine from the extracellular space through the SNAT1 transporter. Then, glutamine is hydrolyzed to glutamate and ammonia by glutaminase. Glutamate is packed into synaptic vesicles and released during neurotransmission. The glutamate is cleared from the synaptic cleft by astrocytes, employing glutamate transporters GLT-1 and, to a lesser extent, GLAST. Astrocytic enzyme glutamine synthetase catalyzes the reaction of glutamate amidation and generate glutamine. Finally, glutamine is released from astrocytes via the SN1 transporter.

The healthy brain utilizes glutamine to synthetize glutamate, the prevailing activatory neurotransmitter. Since neurons are unable to synthesize either the neurotransmitter glutamate or γ-aminobutyric acid (GABA) from glucose, glutamate synthesis involves neuron–astrocyte cooperation termed the glutamine–glutamate cycle (Figure 1) [18].

Glutamate is synthetized in glutamatergic neurons by mitochondrial enzyme glutaminase (GA; glutamine aminohydrolase) (EC 3.5.1.2), which hydrolyses glutamine transported into the neurons by the system A transporter SNAT1 (Slc38a1). This reaction (glutamine + H_2_O → glutamate + NH_3_) is the first step of glutaminolysis (i.e., stepwise conversion of glutamine into glutamate, consecutively transformed into αKG, an intermediate of the TCA cycle). After glutamate is released from neurons, it is taken up from the synaptic cleft by astrocytes, employing glutamate transporters (EAATs), Glast (Slc1a3), or GLT1 (Slc1a2). In astrocytes, glutamate is amidated to form glutamine, by the enzyme glutamine synthetase (GS; glutamate-ammonia ligase; GLUL) (EC 6.3.1.2), catalyzing the reaction glutamate + ATP + NH_3_ → glutamine + ADP + phosphate. Then, glutamine is transported out by the system N transporter, SN1 (Slc38a3). Finally, it is taken up from the synaptic cleft by neurons (closing the cycle) or, marginally, leaks into the blood vessels [19,20].

GS plays a key role in nitrogen metabolism, using ammonia derived from amino acid degradation. In the brain, GS is expressed exclusively in astrocytes [21], which makes them solely responsible for both glutamine synthesis and ammonia detoxification. Moreover, GS as a part of the glutamine–glutamate cycle, participates in neurotransmitter glutamate recycling, its clearance from the synaptic cleft and synthesis of its precursor. In total, 70% of the neurotransmitter pool of glutamate is derived from astrocytic GS-generated glutamine [22].

Human GA is encoded by two genes: *GLS* encoding the kidney-type KGA and GAC isoforms, and *GLS2*, encoding the liver-type isoforms, GAB and LGA. The *GLS* gene expression may be modulated by such oncogenes as *MYC*, *Rho GTPases*, and *Notch*, while the *GLS2* gene was reported to be regulated by p53 (references in [23]). Both *GLS* and *GLS2* products are activated by phosphate (therefore the previous name: phosphate-activated glutaminase, PAG); however, GLS2 is activated by lower concentrations of phosphate and to a lesser extent than GLS. Secondly, GLS2 is activated and GLS inhibited by ammonia. Thirdly, GLS2 has high whereas GLS has low Km for glutamine [24]. GLS is mainly active in the kidney, brain, and liver [25], and isoenzymes’ expression is tissue specific. In the brain, GLS isoforms predominate while GLS2 are less abundant [26]. The common view that *GLS* is expressed only in neurons was recently challenged by Cardona et al. [27], who showed both KGA and LGA/GAB expression and in situ activity in human and rat hippocampal, cerebral, and cerebellar astrocytes. KGA was almost entirely mitochondrial whereas GAB/LGA was detected in mitochondria and nuclei.

## 4. Glutamine Addiction in Gliomas

Although glutamine is considered a non-essential amino acid and its concentration in blood is high (~550 μM) [28], in pathological conditions of increased demand, it becomes conditionally essential, as in glutamine-addicted neoplasms. Wise et al. [29] showed that SF188 glioma cell line viability decreased to zero when glutamine was absent in the culture medium. The glutamine addiction of those cells was evident despite the presence of high levels of glucose [29].

The maintenance of high-rate pyrimidine synthesis may be limiting for gliomas, and targeting this pathway resulted in decreased viability of GBM stem cells [30]. Additionally, decreased nutrient availability leads to activation of the catabolic process to provide substrates for biosynthesis. Therefore, the TCA cycle intermediates, utilized as macromolecules precursors, must be resupplied. The anaplerotic pathways serving this purpose are pyruvate carboxylation and glutaminolysis. However, the reaction catalyzed by pyruvate carboxylase (PC), generating oxoloacetate (that condenses with acetyl-coA to form citrate, which enters the TCA cycle) from pyruvate, is downregulated in most neoplasms [4]. From the opposite perspective, in contrast to SF188 GBM cell line with originally low PC activity, cells with experimentally upregulated PC became glutamine independent and silencing GA expression did not compromise their growth [31]. Therefore, the glutamine-derived αKG remains a major way to supply the TCA cycle.

The glutamate dehydrogenase (GLUD)-catalyzed reaction, producing αKG and ammonia, is reversible, but in neoplasms, αKG is mostly synthetized. The reaction may be activated by low energy levels (ADP) and by leucine and mTOR, which makes GLUD willingly exploitable by gliomas [32]. Aminotransferases synthetize αKG without releasing ammonia. In the CNS, both alanine aminotransferase (glutamate-pyruvate transferase; GPT) and aspartate aminotransferase (glutamate-oxoloacetate transaminase; GOT) operate [33].

The metabolic fate of glutamine-derived glutamate is, aside from αKG, GSH and lactate. The GSH is synthetized by glutamate-cysteine ligase and both its synthesis and degradation is controlled by nuclear factor erythroid 2-related factor 2 (NRF2) transcription factor [34]. For the reason that GSH is a potent antioxidant and the main factor responsible for treatment resistance in gliomas or other neoplastic cells, therapeutic attempts were aimed at GSH depletion by inhibiting the X_C_^−^ transporter, responsible for counter-transport of glutamate and cysteine-a substrate-limiting GSH synthesis [35,36].

Lactate is of special importance for tumor pathophysiology and for gliomas in particular due to specific CNS features. As proposed by Pellerin et al. [37], in physiological conditions, astrocytes take up glucose via the Glut1 transporter and metabolize it to lactate and release it through the Slc16a3 (MCT4) transporter [38]. Then, neurons take up lactate via the Slc16a7 (MCT2) transporter and use it, and not glucose, as a direct and primary source of energy. Of note, the scope of the lactate shuttle contribution to neuron energetics remains debatable [39]. Even though glutamine serves anabolic purposes, and glutamine-derived glutamate constitutes a substantial carbon and nitrogen pool for augmented synthesis of nonessential amino acids and nucleotides, respectively, the research indicates that in neoplasm, a prevailing amount of glutamine is converted to lactate and released from the cell, mirroring the glycolytic Warburg effect. This seemingly futile process involves a step of oxidative decarboxylation of malate to pyruvate, CO_2_, and NADPH that serves as an unneglectable source of reducing power, necessary to drive glycolysis by GAPDH [40]. Secondly, lactate is a potent signaling molecule that has now been implicated in multiple steps in tumorigenesis, acting via hypoxia-inducible factor-1 (HIF-1) and vascular endothelial growth factor (VEGF) [41].

**Figure 2:**
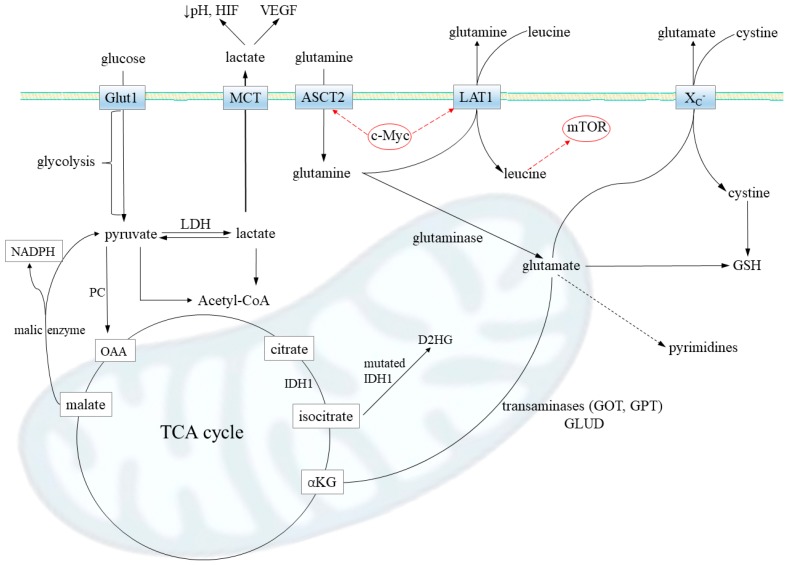
Metabolic pathways contributing to glutamine addiction in gliomas. Firstly, glutamine transport via Slc1a5 transporter (ASCT2) is increased in gliomas. The promoters of the Slc1a5 gene are under transcriptional control of oncogenic transcription factor c-MYC, which upregulates its expression. A substantial portion of intracellular glutamine is released from the cell via transporter LAT1 (Slc7a5; also upregulated by c-MYC) in exchange for extracellular leucine, an essential amino acid. The glutamine–leucine shuttle directly activates mTOR signaling, leading to increased protein synthesis. That way, an abundance of glutamine further accelerates the glioma anabolism. When in the cell, glutamine may be converted by glutaminase to glutamate, which is then metabolized to (i) alpha-ketoglutarate (αKG) (by transaminases GPT and GOT or glutamate dehydrogenase, GLUD) and serves for tricarboxylic acid (TCA) cycle anaplerosis; the reaction catalyzed by pyruvate carboxylase (PC), generating oxaloacetate (OAA) from pyruvate, is downregulated in most neoplasms; (ii) oncometabolite D-2-hydroxyglutate (D2HG) in the case of mutations in IDH1 enzyme, leading to a loss of proper enzymatic activity; iii) antioxidant glutathione (GSH), responsible for the treatment resistance; transporter XC- performs antiport of glutamate and cystine, which is a limiting substrate in GSH synthesis; and iv) lactate, which may be a source of energy when glutaminolysis takes place and the malate shuttle operates (malic enzyme synthetize lactate precursor, pyruvate, and NADPH) but also modulate tumor invasiveness via vascular endothelial growth factor (VEGF) and hypoxia-inducing factor (HIF); lactate was shown to stabilize HIF and then transactivate c-Myc in a pathway that mimics a response to hypoxia. In concordance to high lactate release observed in glioma, the lactate dehydrogenase (LDH), responsible for lactate synthesis, has been often reported to be upregulated. Last, but not least, glutamine availability is a limiting step for pyrimidine synthesis that highly proliferative cells, such as gliomas, need to be kept high.

Aside from αKG, glutamine may be converted via glutamate to proline [42], which gained attention as a modulatory molecule in cancer biology [43]. Proline may be synthetized by pyrroline 5-carboxylate reductase (PYCR) in a reaction linked to NADPH oxidation, which makes proline synthesis a likely response to increased NAD^+^ demand, which may occur in hypoxia. c-Myc and PI3K upregulate proline synthesis [43]. Enhanced proline synthesis by PYCR, supporting mitochondrial redox homeostasis, was reported in an oligodendroglioma cell line with an *IDH1* mutation [44]. Following synthesis, proline may be catabolized by the proline oxidase (proline dehydrogenase; POX/PRODH), a putative tumor suppressor. The overexpression of proline oxidase decreased the intracellular levels of proline, glutamate, and glutamine, and moderately decreased growth of the U87MG GBM cell line [45]. The gene encoding POX/PRODH, *PRODH*, was found to be decreased in GBM [46], and increased proline levels were reported in the cerebrospinal fluid of patients with glioma [47]. Interestingly, Grinde et al. [48] showed that breast cancer cell-derived xenograft used glutamine for proline synthesis and reacted to glutaminase inhibition with growth inhibition, whereas the subtype utilizing glutamine just for glutamate and lactate synthesis was nonresponsive [48]. The metabolic pathways, where glutamine directly or glutamine-derived compounds contribute to glioma pathology and render them glutamine addicted, are briefly indicated in Figure 2.

### 4.1. Glutamine Addiction in the Context of Glucose Addiction

The oversupply of glucose and increased aerobic glycolysis (Warburg effect) result from oncogenic mutations. The stabilization of HIF-1α, p53 loss, upregulation of PI3K and its downstream AKT kinase and mTOR pathway, and overexpression of c-Myc transcription factor are the basic mechanisms of genetic adaptations of brain neoplasms leading to glycolysis enhancement and glucose addiction [49,50,51,52,53]. The c-Myc transcription factor’s overexpression in 293T and U87MG GBM cell lines resulted in increased levels of glycolytic intermediates (glucose- and fructose-6-phosphate, pyruvate, lactate) and decreased cell viability in glucose-free media. The glucose addiction of cells, induced by c-Myc upregulation rendered them susceptible to glycolysis inhibition by nicotinamide phosphoribosyltransferase (Nampt) inhibitor [54]. In case of c-Myc, the consequences of its activation are also relevant for glutaminolysis, raising the possibility of concomitant glucose and glutamine addiction. The c-Myc was found to upregulate GA activity (see Section 3) and glutamine uptake via the ASCT2 transporter (reviewed in [55]).

The functional link between glycolysis and glutaminolysis was described by Smith et al. [56]. In colon cancer cell lines, oncogenesis involves upregulation of LDH isoform A (at the expense of LDHB) and glutamate-pyruvate transaminase 2 (GPT2). The glutamine-derived glutamate and glucose-derived pyruvate are substrates for GPT2 that synthetizes αKG. By GPT2 upregulation, the anaplerotic replenishment of the TCA cycle is possible; otherwise, it is impaired by augmented pyruvate conversion to lactate. In other words, the Warburg effect, manifested as increased lactate release, drives glutamine addiction in order to maintain the TCA cycle function.

In gliomas, glucose addiction that led to glutamine addiction was shown in GBM cell lines in vitro and in xenograft models [57,58]. When grown in glucose-deficient medium, the SF188 cells, characterized with c-Myc-associated enhanced glutamine metabolism, developed an adaptation manifested as increased activity of GLUD, allowing TCA anaplerosis. The interrelation of glyco- and glutaminolysis was emphasized by the fact that suppressing Akt signaling (i.e., suppressing glycolysis) also activated GLUD [57]. Similar glucose–glutamine relatedness was revealed by Tanaka et al. [58]. Treatment with mTOR inhibitors suppressed glucose consumption and increased glutaminase expression and the activity to take over and sustain the viability of GBM cell lines (U87MG, U251MG, LN229, T98G, A172) in vitro and in U87MG xenografts in mice [58].

### 4.2. Metabolomic Studies in Gliomas

In search of the mechanism responsible for the favorable phenotype of neoplasms, enabling them to successfully compete with healthy cells, metabolomics have been employed in addition to genomic and proteomic studies. In contrast to the latter ‘omics’, metabolomics allows for direct and quantitative assessment of biochemical activity and the functional state of cells or tissues, providing the best representation of their molecular phenotype. In terms of glutamine addiction, natural changes in glutamine, glutamate, and αKG levels, correlating with changes in the relevant enzymes’ and transporters’ activity, should be expected.

Cuperlovic-Culf et al. [59] investigated nine human glioma cell lines’ (A172, BS149, Hs683, LN18, LN229, LN319, LN405, U343MG, U373) metabolome. The ^1^H NMR spectroscopy analysis of the intracellular levels of metabolites allowed four groups with distinct metabolic profiles to be distinguished. However, glutamine and glutamate levels were increased in two groups and glutamate in one. The increased amino acids levels correlated with the expression of relevant transporters.

The metabolic heterogeneity of GBM in terms of glucose/glutamine utilization was also described by Oizel et al. [60]. The metabolic phenotype (glucose, glutamine, alanine, and aspartate consumption and production; mitochondrial oxidation; and extracellular acidification as measures of the glycolytic rate) of eight primary GBM cultures was determined. The analysis revealed two GBM subtypes, termed “glutamine-high” and “glutamine-low”. The glutamine-high type was characterized by a high glutamine consumption rate and the ability to convert glutamine (and other nutrients as well) to NADH. Inhibition of the glutamine metabolism decreased proliferation in vitro and in mice xenografts of glutamine-high (despite their ability to utilize other nutrients) and not glutamine-low cells. Importantly, the glutamine-high cell subtype lacked expression of CD133, associated with stem cells, and displayed a mesenchymal signature. In contrast, glutamine-low cells, exhibiting a strong dependency on glucose, expressed the CD133 marker [60]. Similar observations were made by Tardito et al., who generated paired populations of differentiated cells (DIFF) and GSCs from patient-derived GBM cells. While DIFF proliferation was attenuated in the absence of glutamine, GSCs grew independently of supplementation with this amino acid. GSCs displayed a markedly higher expression of GS compared to DIFF. Furthermore, most human GBM, as well as GS-proficient, GBM xenografts withdrew carbons from the TCA cycle to synthesize glutamine via GS [61].

Marin-Valencia et al. studied metabolites in tumors formed by patient-derived genetically diverse GBM cells implanted into mice without prior adaptation to the cell culture. These tumors avidly consumed glucose and accumulated a large pool of glucose-derived glutamine but exhibited minimal glutaminolysis. While GBM cells isolated from these tumors were highly dependent on glucose, they did not require glutamine for survival. These cells expressed significant amounts of c-Myc, PC, and GS, but only traces of GLS, and produced glutamine from glucose [49].

In another study, the culturing of patient-derived GBM cells under restricted glucose increased the expression of several stem cell markers (like Oct4, Nanog, Sox2, CD133) and decreased levels of the astrocyte differentiation marker, glial fibrillary protein (GFAP). Culturing in low glucose increased the ability of these cells to form neurospheres and in vivo tumor growth. Further analysis also revealed that brain tumor-initiating cells (BTICs) preferentially survived restricted glucose conditions and that non-BTICs could adapt to these conditions through BTIC marker expression. Moreover, BTICs displayed elevated levels of glucose transporter Glut3 to permit preferential glucose uptake and targeting ogGlut3 BTICs growth and tumorigenic potential [62]. By contrast, Vlashi et al. showed that GSCs and progenitor cells were less glycolytic and produced less lactate but contained higher ATP levels than differentiated glioma cells. Furthermore, GSCs tolerated inhibition of glycolysis or oxidative phosphorylation well [63].

The reasons for the discrepancies between the abovementioned results may be caused by the use of different cell subtypes and the methods of their isolation. However, they could also reflect a great capacity of BTICs and GSCs for shifting their metabolomics profiles in order to adapt and survive [64]. A high level of GSCs heterogeneity has recently been documented by Shibao et al. According to findings, while murine GSCs are not completely dependent on either glycolysis or oxidative phosphorylation alone, they are able to adopt either predominant metabolic phenotype in an immunocompetent environment. At least a subgroup of GSCs display metabolic plasticity, which contributes to the response to energy pathway inhibitors [65].

The level of metabolites was also measured in human gliomas with two approaches: direct measurement in the brain [66,67] and in the plasma [68,69] or cerebrospinal fluid [47]. Kallenberg et al. reported that glutamine levels, assessed with magnetic resonance, were higher in the hemispheres of patients with GBM than in healthy controls [66]. Wibom et al. measured the level of metabolites in microdialysates obtained from the tumor, brain tissue adjacent to the tumor (BAT), and reference tissue (abdomen area) [67]. Among other changes, decreased glucose levels and increased glutamine and glutamate in the tumor and/or BAT were observed. Interestingly, treatment with radiotherapy evoked changes in some of the identified compounds’ level, including glucose (increase) and glutamine and glutamate (decrease). The authors hypothesized that this reversing trend in the glucose and glutamine concentration may reflect reduced proliferation whereas increased glutamate may be a result of a spillover due to cell damage. However, Mörén et al. reported the opposite: an increase of glutamine levels in the tumor and BAT following radiotherapy in patients with high-grade gliomas [70].

The measurement of metabolites in cerebrospinal fluid or plasma is aimed at finding a distinctive metabolic footprint that might serve as a biomarker, diagnostic, or prognostic tool or as a guide for monitoring of the course of treatment. As such, the results are less informative in terms of glucose/glutamine addiction. Nonetheless, Kelimu et al. identified the metabolic profile of 20 plasma metabolites, allowing for differentiation between healthy and glioma-bearing patients [68]. Similarly, the metabolite profile of the cerebrospinal fluid of glioma patients differed from control enrichment in the TCA cycle, or tryptophan pathway metabolites indicated malignancy or inflammatory processes, respectively [47]. Further, Zhao et al. reported that five metabolites detected in plasma (uracil, arginine, lactate, cystamine, and ornithine) significantly differed between high- and low-grade glioma patients, and three of those were characteristic for gliomas with *IDH1* mutants [69].

Clearly, the metabolic state of particular populations of glioma cells, including GSCs, is still far from being understood. The abovementioned studies indicate a high metabolic heterogeneity of glioma subtypes as well as metabolic flexibility of GSCs. Moreover, they also indicate that the tumor microenvironment may significantly influence the metabolism of these cells.

## 5. Targeting Gln Addiction in Brain Tumors

A study by Dranoff et al. [71] showed that exogenous glutamine was limiting for the proliferation of some GBM cell lines, while the other GBM and medulloblastoma cell lines grew in the absence of this amino acid and this variation most likely resulted from the differences in the activity of GS. Glutamine analogs, 6-diazo-5-oxo-L-norleucine (L-DON) and acivicin, reduced proliferation of GBM and medulloblastoma cells, and GS inhibitor, methionine sulfoximine (MSO), displayed antiproliferative properties against medulloblastoma cells with high GS activity. These results suggested that suppression of glutamine metabolism may represent an attractive therapeutic strategy for the treatment of brain tumors [71]. The same group also examined the properties of other glutamine antimetabolites in GBM and medulloblastoma cells. The most promising results were obtained for L-asparaginase, presenting glutaminase activity as well as actinomycin D and 5-azacytidine, both modulating GS activity. In two drug combination studies, synergism was detected between MSO and DON or acivicin, and complementary inhibition was observed when the cells were treated with acivicin and L-asparaginase [72]. Apart from the promising antitumor activity, all the compounds mentioned above present undue neurotoxicity [73,74], therefore their clinical use is limited. Notwithstanding these limitations, the targeting of glutamine metabolism in brain tumors has received increasing attention in the last years.

### 5.1. Suppressing Gln Uptake

Functional studies suggest that glutamine transport in the rat glioma C6 cell line is mediated mainly by the neutral amino acid uptakesystem ASC [75]. Human GBM and AA presented significantly increased levels of ASCT2 transcript as compared to the non-tumorigenic brain tissues, and similar ASCT2 upregulation was observed in high-grade glioma patient-derived cell cultures as well as brain metastases [76]. The same study revealed overexpression of SNAT3 mRNA in samples derived from AA and GBM compared to both non-tumorigenic brain tissues and brain metastases, and a high abundance of SNAT3 protein was observed in GBM specimens. Of note, the expression of this transporter was negligible in primary cultures derived from high-grade gliomas [76]. The significance of SNAT3 upregulation in high-grade gliomas remains unknown. Preliminary findings from our laboratory suggest that silencing of *SNAT3* neither reduces total glutamine uptake nor significantly alters the proliferation of human GBM T98G cells, which, in contrast to the primary cells derived from patients with GBM, express considerable amounts of *SNAT3* transcript (unpublished data). The lack of an influence of *SNAT3* silencing on glutamine uptake most likely results from the relatively low involvement of this transporter in glutamine transport in glioma cells [75,77]. An effect of the modulation of ASCT2 on the phenotype of glioma cells has not been examined so far. Taking into account its crucial role in glutamine uptake in glioma cells, it is tempting to speculate that ASCT2 inhibition could suppress the proliferation and/or migration of these cells. Of note, several studies have shown that suppression of ASCT2 curbs the growth of cancer cells of different origins both in vitro and in vivo (reviewed in [78]).

### 5.2. Modulation of GAs

Increased levels of GLS isoforms found in human GBM tissues [79] make these proteins attractive targets for anti-glioma therapy. The first study devoted to this issue showed that inhibition of GLS, either with siRNA technology or with a specific allosteric inhibitor bis-2-(5-phenylacetamido-1,2,4-thiadiazol-2-yl)ethyl sulfide (BPTES), slowed the proliferation of human GBM D54 cells expressing mutated *IDH1*. Treatment with BPTES elevated levels of glycolytic intermediates, indicating alterations in the glycolytic flux [80]. The *IDH* mutant gliomas (patient-derived glioma stem-like cells and human oligodendroglioma cell line grown in vitro and in xenografts) were also shown as being specifically sensitive to GLS inhibition by CB-839. The glutamate deficiency due to BCAA transaminases inhibition by D2HG was potentiated by the GA inhibition and resulted in lowering of the GSH level, which rendered the gliomas susceptible to experimental treatment, including oxidative stress and radiation [81].

In human GBM cell lines, LN229 and SF-xL, silencing of *GLS* significantly reduced, although not eliminated, proliferation, ability to form colonies, and growth of subcutaneous xenografts. Of note, *GLS* suppression induced a compensatory anaplerotic mechanism dependent on PC [31]. Further analysis showed that *GLS* silencing reduced the GSH-dependent antioxidant capacity of LN229 and SF-xL cell lines and induced apoptosis, most likely mediated by mitochondrial dysfunction [82]. Reactive oxygen species (ROS) generation synergized with *GLS* silencing to suppress malignant properties of GBM cells. Of note, the combination of *GLS* silencing and arsenic trioxide (ATO) treatment reduced the level of c-Myc and anti-apoptotic Bcl-2, as well as increased the level of pro-apoptotic Bid [82]. A recent study not only confirmed our previous notion that *GLS* expression is significantly elevated in GBM tissues relative to the non-tumorigenic brain but also indicated increased glutamine and glutamate levels in GBM compared to the non-tumorigenic brain tissues. Moreover, the level of *GLS* was frequently elevated in tumor samples compared to the level of *GS*, suggesting a metabolic flux from glutamine to glutamate for the high rates of glutamine catabolism. Inhibition of mTOR resulted in an increased intracellular glutamate level and upregulation of *GLS* in GBM U87MG and, to a greater extent, U87/EGFRvIII cells. Similarly, *GLS* upregulation caused by mTOR inhibition was found in U87/EGFRvIII xenograft tumors. Furthermore, combined GLS and mTOR inhibition resulted in massive GBM cell death and tumor growth inhibition in a xenograft model, underscoring the importance of compensatory glutamine metabolism in promoting GBM resistance to mTOR inhibitor treatment [58]. Another recently published study demonstrated that treatment with a GLS-selective inhibitor, compound 968, suppressed the growth of patient-derived GBM cells with an inhibited Notch pathway. Notch blockade diminished the expression of *GLS*, as well as levels of glutamine and glutamate, indicating that the antiproliferative effect of Notch inhibition might be partially mediated by alterations in the glutamine/glutamate cycle [83].

While GLS appears to play a pro-oncogenic role in most cancer types, the role of GLS2 in carcinogenesis is much less understood and seems to be tissu and context specific (reviewed in detail in [84]). Our previous study documented a lack or only traces of *GLS2* transcript in GBM tissues [79,85], therefore we hypothesized that this phenomenon may have implications for the physiology of glia-derived tumors. To address this issue, human GBM T98G cells, showing no measurable *GLS2* transcript level, were transfected with the full GAB cDNA sequence. The transfectant (herein referred to as TGAB) presented a significant decrease of survival, proliferation, ability to form colonies, and migration compared to the sham-transfected or wild-type counterparts [86]. Nuclear localization of GAB and its ability to interact with PDZ proteins put forward the hypothesis that GAB could be a multifaceted protein directly or indirectly influencing transcription [87]. We therefore compared the transcriptomes of TGAB cells and controls. Microarray analysis revealed a set of genes whose expression was significantly up- or downregulated in TGAB cells compared to the controls. Some of these genes encode proteins related to oncogenesis, e.g., MGMT [86]. Given the role of MGMT in cell resistance to treatment with alkylating agents, we speculated that the downregulation of *MGMT* found in TGAB cells might sensitize these cells to such a treatment. Indeed, TGAB cells appeared to be significantly more susceptible to TMZ and the other alkylating drug, carmustine, compared to the controls. Transfection with a GAB sequence diminished not only the level of *MGMT* transcript but also MGMT protein and its activity, most likely contributing to the increased sensitivity to alkylating agents [88]. Our recent study showed that transfection with a GAB cDNA sequence reduced viability, proliferation, and the ability to form colonies of the other human GBM cells, LN229 and U87MG, varying with respect to the *TP53*, *PTEN*, and *MGMT* status and tumorigenic potential. Moreover, the cells transfected with a GAB sequence (termed LNGAB and UGAB, respectively) appeared to be more sensitive to TMZ treatment compared to the controls. As each of the parental cell lines (LN229 or U87MG) does not express *MGMT* [89], an MGMT-independent mechanism had to be involved in the increased susceptibility to TMZ observed in LNGAB and UGAB cells. A previous study revealed that TGAB cells were more sensitive to oxidative stress compared to the controls [87]. In agreement with this observation, an increased sensitivity to H_2_O_2_ was found in LNGAB and UGAB. Further analysis showed that in all three cell lines’ transfection with a GAB sequence diminished the phosphorylation level of AKT, and this phenomenon contributed to the increased susceptibility of GBM cells towards H_2_O_2_ [14]. These data are in agreement with the previous notion that negative regulation of the PI3K/AKT pathway mediates the role of GAB in the suppression of hepatocellular carcinoma growth [90].

Increasing evidence showing that the aggressive GBM phenotype could be reversed either by *GLS* inhibition or *GLS2* overexpression led us to test whether or not the combination of these two procedures would increase the inhibition of survival of GBM cells elicited by individual manipulations. We therefore knocked down *GLS* in T98G (not expressing GLS2) or TGAB (previously transfected with GAB cDNA). In both T98G and TGAB cell lines, silencing of *GLS* with siRNAs targeting KGA or GAC reduced cell viability and proliferation in a sequence-dependent degree. Of note, TGAB cells treated with anti-GLS siRNA presented lower viability and proliferation compared to T98G so treated, although the proportional decrease of these parameters evoked by *GLS* silencing was in T98G than TGAB cells [91].

Taken together, a growing body of evidence suggests that GLS2 displays a glioma-suppressive function, as it has also been postulated in the case of liver and colon cancers [90,92,93]. Clearly, further analysis is needed to unravel the detailed molecular mechanism underlying this phenomenon. The studies on the role of GLS2 in GBM performed so far exclusively used GBM cell lines, therefore the results should be interpreted with caution. The other, more clinically relevant models, like patient-derived GBM cells and patient-derived xenografts, will have to be employed in future research.

### 5.3. Targeting Glutamate Transport

Studies performed on primary human glioma cultures and GBM cell lines clearly showed remarkably lower rates of glutamate uptake in tumor cells compared to normal astrocytes. Moreover, GBM cells release large amounts of this amino acid into the culture medium. It is noteworthy that exposure of cultured neurons to GBM-conditioned medium elicits [Ca^2+^]*_i_* elevations followed by neuronal death. Furthermore, coculturing of neurons and GBM cells, either with or without direct contact, causes neuronal damage. GBM-induced neuronal death could be prevented by treating neurons with antagonists of NMDA (*N*-methyl-d-aspartate) or AMPA (α-amino-3-hydroxyl-5-methyl-4-isoxazole-propionate) receptors or by depletion of glutamate from the medium [94].

An impaired glutamate transport in GBM cells most likely results from alterations in the expression, activity, and cellular localization of glutamate transporters. The expression of GLT-1 is abundant in rat astrocytes and human non-tumorigenic brain tissues, but human GBM cell lines and tissues lack this protein [95]. This finding was confirmed by microarray analysis, which showed decreased GLT-1 expression in high-grade glial tumors compared to low-grade astrocytomas and the normal brain. Overexpression of GLT-1 inhibited proliferation and induced apoptosis in human GBM cell lines as well as suppressed tumor formation in mouse xenografts, suggesting that the loss of this transporter may contribute to the aggressive phenotype of glioma [96].

Data on the expression pattern of another glutamate transporter, GLAST, in gliomas are less consistent. Ye et al. detected GLAST expression on the surface of astrocytes but in the nuclei of GBM cultured cells. A similar mislocalization of this transporter to the nuclei was observed in human GBM tissues, suggesting that this altered cellular localization of GLAST may partially contribute to inefficient glutamate uptake [95]. However, a recent study showed an intense expression of GLAST in high-grade gliomas compared to low-grade tumors and, in contrast to the previous report, this protein was mainly localized in the plasma membrane. In a group of 36 patients with glioma, the strong or moderate GLAST expression significantly correlated with lower overall survival, supporting a potential role of GLAST as a prognostic marker for GBM. Both murine glioma GL261 and human GBM stem-like cells expressing high levels of GLAST (herein referred to as GLAST^high^ cells) appeared to be significantly more aggressive in terms of survival compared to the low GLAST-expressing cells (herein referred to as GLAST^low^ cells) after intracranial injection into mice. Moreover, tumors generated from GLAST^low^ cells presented a reduced invasive ability compared to gliomas formed by control cells. Silencing of *GLAST* with shRNA inhibited the proliferation and migration of GSCs and the progression of tumors generated by GSCs, resulting in prolonged survival. The amounts of intracellular glutamate were significantly lower in both human and murine GSCs compared to astrocytes. By contrast, the extracellular level of this amino acid in the medium of GSCs was higher than in astrocytes, and its significant reduction was observed in GSCs with silenced *GLAST*, suggesting an involvement of this transporter in the release of glutamate by GSCs. When GLAST^high^ cells were injected into mice, the glutamate concentration increased even before tumor appearance at both the injection site and in the contralateral hemisphere and was higher than in GLAST^low^ tumors. At later time points, gliomas from GLAST^high^ cells grew in two hemispheres. Moreover, a decreased glutamate level was found within the tumor mass, while its highest content was detected in peritumoral regions contralaterally, supporting the role of glutamate in glioma cell infiltration into the opposite hemisphere. Treatment with UCPH-101, a specific inhibitor of GLAST, caused apoptosis and necrosis of glioma cells but did not affect astrocytes. Intratumoral injection of a single dose of UCPH-101 significantly decreased tumor mass and increased the survival of GL261 glioma-bearing mice without causing toxicity. These findings not only underscore the involvement of GLAST in glutamate trafficking in gliomas but also indicate that this transporter may be a new therapeutic target [97]. Indeed, a recent study showed that genetic or pharmacologic inhibition of GLAST1 markedly sensitized several cancer cell lines to inhibitors of the electron transport chain (ETC). In GBM tissues, steady state levels of aspartate, which is also transported by GLAST1, negatively correlated with markers of tumor hypoxia, indicating that tumor hypoxia is sufficient to inhibit ETC and aspartate synthesis in vivo [98].

It is noteworthy that immunization with GLAST-derived peptides significantly increased the survival of GL261 glioma-bearing mice by delaying or even abolishing tumor growth. The lack of toxicity following immunization with GLAST-derived peptides could be attributed to the recruitment of the activated immune cells to the tumor site. The authors therefore proposed that GLAST might be a glioma antigen against which immune response could be induced without severe side effects [99].

The study by Ye et al. revealed that in GBM cell lines, over 50% of glutamate transport was mediated by a cysteine-glutamate exchanger, system x_c_^−^ [95]. System x_c_^−^ (SXC) consists of a catalytic light chain (xCT or SLC7A11) and a regulatory heavy chain (4F2hc or CD98). While xCT confers transporter function and substrate specificity, CD98 is essential for membrane localization of the transporter [100]. Recent findings indicate that xCT is directly phosphorylated by mTORC2, which in turn inhibits the activity of this transporter. Silencing of mTORC2 or pharmacological mTOR kinase inhibition promotes glutamate secretion and cysteine uptake, linking growth factor receptor signaling with glucose and amino acid metabolism [101].

High expression of both xCT and CD98 subunits was detected in GBM cell lines and tissues [102]. Although *xCT* silencing with siRNA did not alter the proliferation of glioma cells, it massively reduced glutamate secretion. A similar effect was achieved when glioma cells were treated with 50 μM S-4-carboxyphenylglycine (S-4-CPG), an xCT inhibitor, and the higher concentrations of this compound inhibited glioma cell proliferation. Conditioned media from glioma cells applied to brain slices induced massive neuronal degeneration, while conditioned media from xCT-silenced cells induced only sparse neuronal death. The implantation of xCT-silenced glioma cells into brain tissues resulted in significantly lower cell death compared to the wild-type glioma cells. Moreover, rats with xCT-silenced glioma cells implanted into brains presented a delayed onset and progression of neurological deficits, smaller perifocal edema, as well as prolonged survival compared to the animals with wild-type tumors. Likewise, a delay in the onset and progression of neurological deficits resulting in prolonged overall survival was observed when S-4-CPG was administrated intrathecally in rats with implanted glioma cells [102].

The abovementioned findings clearly present an important role of xCT in glioma-induced neuronal death and are in agreement with previous results documenting high expression of both subunits of SXC in human GBM cell lines and biopsies as well as an inhibitory effect of S-4-CPG on GBM cells’ proliferation [103]. More importantly, the study by Chung et al. showed that pharmacological inhibition of SXC with sulfasalazine (SAS), approved by the Food and Drug Administration to treat inflammatory bowel disease, caused a selective, apoptotic, caspase-mediated cell death of GBM cells in vitro and in a xenograft model of GBM [103]. Further detailed analysis not only confirmed the overexpression of SXC in human GBM cell lines and tissues but also clearly showed the involvement of this transporter in the release of glutamate from GBM cells. Moreover, this study confirmed a crucial role of released glutamate in promoting GBM cells’ migration and invasion both in vitro and in vivo as well as provided proof-of-principle evidence for the utility of SAS to inhibit glioma invasion [104].

The findings mentioned above, supported by the significant increase in peritumoral glutamate detected in GBM patients [105,106], put forward a hypothesis that glutamate released from gliomas activates neuronal receptors, which in turn underlie seizures in the vicinity of the tumor presented by most glioma patients, and precede excitotoxic cell death [107]. Moreover, these data led to the introduction of SAS to clinical studies for patients with malignant gliomas. In a group of 10 patients with advanced recurrent anaplastic astrocytoma or GBM, treatment with SAS as a single agent did not affect tumor growth. As an interim analysis revealed a high incidence of serious side effects as well as a lack of efficacy, this clinical trial (ISRCTN45828668) was terminated [108]. However, this trial enrolled a population of neurologically unfavorable patients with advanced multiply treated disease. A later study showed that glutamate released by GBM cells through SXC activated glutamate receptors on peritumoral neurons, leading to neuronal hyperexcitability and epileptic activity. SAS treatment reduced the epileptic event frequency in tumor-bearing mice, suggesting that this drug could be considered as an adjuvant treatment to ameliorate peritumoral seizures associated with glioma [109]. In another study, the combination of SAS and radiation increased DNA double-strand breaks and increased glioma cell death. Furthermore, SAS and gamma knife radiosurgery synergistically prolonged survival in nude rats harboring human GBM xenografts, compared with controls or either treatment alone, indicating that this drug could enhance the efficacy of current radiotherapies for glioma patients [110].

Recent research identified two sub-groups of glioma patients that either highly express or lack expression of xCT. The cells isolated from xCT-expressing but not from non-expressing glioma tissues produced excitotoxicity in vivo after intracranial implantation into mice, induced seizures, and reduced overall survival. The correlation between survival and the altered expression of *xCT* was also found in patients with gliomas, as patients with reduced *xCT* expression lived 9 months longer than patients with tumors expressing elevated *xCT* levels. Moreover, in nine glioma patients, the expression of *xCT* positively correlated with glutamate release, which was inhibited with oral SAS [111]. Taken together, these data clearly indicate that glutamate release mediated via SXC significantly contributes to glioma-induced excitotoxicity and epilepsy, which can be modified by SAS administration. While this drug has several weaknesses, like a short half-life or susceptibility to cleavage by gut bacteria, it is still the only approved drug penetrating the blood–brain barrier and targeting SXC. Studies searching for improved SXC inhibitors are currently ongoing [112,113]. Of these, erastin appeared to be a very potent and metabolically stable inhibitor of SXC that significantly suppressed lymphoma growth in vivo [114].

It is worth mentioning that the high level of SXC observed in gliomas allowed assessment of the feasibility of (S)-4-(3-[18F]Fluoropropyl)-L-glutamic acid (18F-FSPG) for imaging orthotopic brain tumors in rats and the translation of this approach in patients with intracranial malignancies. 18F-FSPG is a glutamate analogue and its uptake, mediated by SXC, is associated with cellular responses to oxidative stress and detoxification processes, which are important for tumor progression and resistance to treatment. 18F-FSPG showed good uptake in the tumor itself, and low background uptake in the surrounding normal brain. Potential future applications for this PET tomography radiopharmaceutical may include a diagnostic aid to magnetic resonance imaging (MRI) for diagnosis and treatment planning, evaluation of recsidual/recurrent malignancy, and for surveillance [115].

### 5.4. Modulation of GS and GLUD

Huge amounts of glutamate produced by glioma cells draw attention to the role of enzymes metabolizing this amino acid. One of the first reports dedicated to the expression of GS in brain tumors showed a high level of this enzyme in gliomas but also clearly indicated considerable variation between tumors, suggesting a random loss of GS expression during neoplastic transformation or heterogeneity in their cellular origin [116]. According to the later study, GS was highly expressed in astrocytomas of II–IV grade and in oligoastrocytomas of II and III grade while lower amounts or a lack of this protein were detected in oligodendrogliomas [117]. Variation in the level of GS between GBM tissues was also observed by Rosati and colleagues, who additionally found a correlation between the low level of GS in tumor samples and the presence of epilepsy [118]. Later on, the same group not only confirmed this finding but also found a correlation between absent/low GS expression and longer overall survival of newly diagnosed GBM patients [119].

Whether downregulation of GS mechanistically contributes to glutamate accumulation and seizure generation or other phenotypic parameters of glioma cells remains an open question. While the studies mentioned above indicate that low GS levels could be beneficial for GBM patients, the only experimental study performed so far showed that overexpression of GS resulted in growth arrest and motility suppression of rat C6 glioma cells in an N-cadherin-dependent manner while *GS* silencing enhanced cell motility [120]. Further studies are needed in order to conclude whether GS acts as a pro- or anti-glioma enzyme.

As mention above, glutamate is converted to α-KG by GLUD. Increasing evidence indicates that this enzyme contributes to glioma metabolism and growth. A remarkable increase in activity of GLUD, and only minor elevation of GLS activity, was observed in human GBM SF188 cells under glucose deprivation conditions. GLUD knockdown or treatment with epigallocatechin gallate (EGCG) caused cell death in the absence of glucose, but treatment with analogs of pyruvate or α-KG completely rescued the viability of glucose-deprived cells. Downregulation of the Akt pathway, which facilitates glycolysis, increased GLUD activity, and overexpression of Akt suppressed it, suggesting that Akt regulates GLUD through its effects on glucose metabolism [57]. The results of a later study displayed overexpression of *GLUD1* and *GLUD2* in *IDH1^R132H^* human GBM relative to *IDH1^WT^* GBM. Orthotropic grafts of *IDH1^R132H^* glioma cells with silenced *GLUD1/2* demonstrated a statistically significant reduction in tumor volume relative to the grafts of control cells. Expression of human *GLUD1* did not promote the growth of *IDH1^R132H^* murine glioma progenitors, but GLUD2 rescued the growth of these cultures. In an in vivo model, overexpression of *GLUD2* completely abrogated the negative effect of *IDH1^R132H^* on tumor aggressiveness. Furthermore, the introduction of *GLUD2* into *IDH1^R132H^* cells decreased 2-HG production from glutamine-derived α-KG and increased citrate production from the TCA cycle. Taken together, these results revealed that GLUD2 promotes the growth of *IDH1^R132H^* glioma cells in a manner that is not duplicated by overexpression of *GLUD1* [121].

However, the role of GLUD2 in glioma pathogenesis appears to be more complex and may be context specific. In a very recent study, Franceschi et al. showed a correlation between *GLUD2* expression and the survival of GBM patients. *GLUD2* mRNA and protein levels were significantly higher in patients with a recurrence-free survival (RFS) time longer than 25 months compared to that of patients with an RFS shorter than 6 months. Furthermore, patients with proneural GBM, associated with the most favorable outcome, displayed higher levels of *GLUD2* compared to the patients with the other tumor types. Human GBM T98G cells containing relatively low levels of *GLUD2* presented higher proliferation and the ability to form colonies compared to the other human GBM cell line, U118, expressing considerable amounts of *GLUD2*. Overexpression of *GLUD2* in T98G cells decreased proliferation and motility while the silencing of this gene in U118 cells increased these parameters. *GLUD2* overexpression in T98G cells increased, and *GLUD2* silencing in U118 cells reduced the levels of both mitochondrial and non-mitochondrial respiration as well as ROS levels. In vivo studies using *GLUD2*-injected zebrafish embryos revealed that *GLUD2* overexpression decreased glial cell proliferation without affecting neuronal development [122]. In contrast to the previous notion presenting GLUD2 as an enzyme promoting the growth of at least some glioma cells [121], these results suggest that in some glioma cell types, an enhancement of GLUD2 activity could result in blocked/reduced proliferation. Clearly, detailed analyses are necessary to shed more light on the role of this enzyme in glioma pathogenesis.

### 5.5. Reversing Lactate Effects

The inverted pH gradient (decreased extracellular and increased intracellular pH) is a feature of many neoplasms, including gliomas in the rodent model in vivo [123]. The acidic pH in the tumor and surrounding tissue was shown in human patients [124] and rat glioma in vivo [125,126,127]. The acidic milieu promoted the expression of glioma stem cells markers, their self-renewal, and growth in vitro and in rodent xenografts, in the mechanism involving HIF2a [124]. The common view that extracellular acidification results from increased lactate production and release [128] has been discussed by Garcia-Canaveras et al., who proposed that lactate/H+ symporters (MCT) are actually inhibited by low pH [123].

Nevertheless, increased lactate in the microenvironment has been shown to promote increased glutamine uptake and catabolism by an HIF2- (which is similar to HIF1) [129] and MYC-dependent mechanism, as a self-perpetuating vicious cycle [130]. In different brain tumors, high lactate dehydrogenase (LDH) activity and an abnormal LDH isoform expression pattern was observed by Subhash et al. [131], who analyzed 100 biopsy samples. Additionally, Di et al. [132], investigating clinical samples, confirmed a positive correlation between LDHA expression and glioma grade. Moreover, silencing LDHA expression with siRNA in human GBM cell lines U87MG and U251MG resulted in growth inhibition and enhanced sensitivity to TMZ [132]. Consistent results, confirming that LDHA upregulation results from a lack of cyclin G2-mediated inhibition of LDHA activity, were obtained by Li et al. [133], who also employed the U87 and U251 plus mouse GL261 glioma cell lines. The LDHA inhibition in human GBM stem cells led to cell differentiation and death [134].

Moreover, lactate was found to drive *VEGF* expression, facilitating vascularization and thus the nutrition of tumors. *VEGF* expression is upregulated in GBM and positively correlates with the tumor grade in gliomas [135,136]. It was demonstrated in glioma xenografts [137] and U87MG human GBM cell line [138] that an acidic pH, characteristic for solid tumors, activates the *VEGF* gene promotor and increases its transcription, engaging the ERK1/2 MAPK signaling pathway. Therapeutic anti-VEGF strategies have been implemented, e.g., bevacizumab, which is a VEGF-blocking antibody, was shown to be safe for glioblastoma patients’ treatment, also in combination with other therapeutics (for a review, see [139]).

The therapeutic approaches targeting glutamine addiction are summarized in Table 1.

## 6. Conclusions

The genetic mutations leading to neoplastic transformation affect the key metabolic pathways in gliomas, making them extremely capable at competing with healthy cells. The robust aerobic glycolysis provides unquestionable advantages, i.e., survival in hypoxic conditions and elevated lactate facilitating invasiveness, but imposes a need for the implementation of anaplerotic mechanisms, indispensable for supporting the anabolic and catabolic processes, and intensified in highly proliferative cells. Glutamine addiction becomes a solution. Increased transport provides glutamine as a source of carbon for the TCA cycle, and then it may be redirected via the malate shuttle to become a source of additional energy and to add up lactate. Further, glutamine becomes essential for nucleotide synthesis and augments the GSH redox system capability, enabling gliomas to resist radio- and chemotherapy. Although the abovementioned mechanisms are relatively well recognized, targeting them clinically remains a challenge for medicine, and not only due to drugs’ adverse effects. The reasons for this are the high heterogeneity of those neoplasms in terms of the genetic background and metabolic strategy (e.g., a recent study showed that GSCs are less glycolytic than differentiated glioma cells) on the one hand, and their remarkable plasticity, enabling them to adjust their metabolism to changing growth conditions and nutrient supplies. It inevitably implies the need for combined personalized treatment, based on identification of the genetic profile of each particular glioma case.

## Figures and Tables

**Table 1:** Therapeutic approaches targeting glutamine addiction in gliomas.

Class	Therapeutic Approach	Model	Outcome	Ref.
glutamine analog	L-DON or acivicin	D54MG human GBM cell line; TE671 human medulloblastoma cell line	decreased proliferation	[71]
glutamine depletion	L-asparaginase	D54MG human GBM cell line; TE671 human medulloblastoma cell line	decreased proliferation	[72]
GLS inhibition	BPTES	D54MG human GBM cell line WT and *IDH1^R132H^*	decreased proliferation in *IDH1^R132H^*	[80]
	compound 968	U87MG and U87MG/EGFRvIII human GBM cell line-in vitro and in mouse s.c. xenograft	enhancement of anti-GBM effects of mTOR inhibition	[58]
		patient-derived GBM cells	decreased viability of cells with an inhibited Notch pathway	[83]
	CB-839	human oligodendroglioma cells WT and *IDH1^R132H^* in vitro and in s.c. xenografts	decreased proliferation in *IDH1^R132H^*; increased sensitivity of animals to radiation	[81]
	GLS shRNA	LN229 and SFxL human GBM cell lines-in vitro and in mouse s.c. xenograft	decreased cell proliferation and growth of xenografts	[31]
	GLS shRNA	LN229 and SFxL human GBM cell lines	decreased proliferation and migration, increased sensitivity to oxidative stress	[82]
	KGA or GAC siRNA	T98G human GBM cell line	decreased proliferation	[85]
GLS2 induction	GAB overexpression	T98G, LN229, U87MG human GBM cell lines	decreased proliferation and migration, increased sensitivity to oxidative stress and TMZ	[23,86,88]
glutamate uptake induction	GLT1 overexpression	U251MG, U87MG, U373, and SNB19 human GBM cell lines; U87MG mouse s.c. xenograft	decreased cell proliferation and growth of xenografts	[96]
glutamate transport inhibition	GLAST shRNA	patient-derived GSCs-in vitro and in mouse intracranial xenograft	decreased cell proliferation and migration and growth of xenografts	[97]
	UCPH-101	patient-derived GSCs; GSCs obtained from GL261 mouse GBM cell line-in vitro and in mouse intracranial xenograft	increased apoptosis in vitro; decreased growth of xenografts and prolonged animal survival	[97]
	GLAST peptides	GSCs obtained from GL261 cells in mouse intracranial xenograft	prolonged animal survival	[99]
	xCT siRNA	F98 rat GBM cell line-in vitro and in orthotopic rat xenograft	unchanged cell proliferation; delayed onset and progression of neurological deficits in animals; prolonged animal survival	[102]
	S-4-CPG	U87MG, U373MG human GBM cell lines; F98 rat GBM cell line-in vitro and in orthotopic rat xenograft	decreased cell proliferation; prolonged animal survival	[102]
		D54MG, U87MG, U251MG, STTG1 human GBM cell lines; patient-derived GBM cells	decreased cell proliferation and migration	[103,104]
	SAS	D54MG, U87MG, U251MG, STTG1 human GBM cell lines; patient-derived GBM cells; mouse intracranial xenograft (D54MG cells)	decreased cell proliferation and migration; decreased tumor growth and invasion	[103,104]
		mouse intracranial model (U251MG human GBM cell line and patient-derived GBM cells)	decreased epileptic activity	[109]
		A172, U251MG and LN18 human GBM cell lines; rat s.c, xenograft (patient-derived GBM)	decreased cell proliferation; increased sensitivity to gamma knife radiosurgery in vivo	[110]
		10 patients with AA or GBM	no clinical response in phase I/II clinical trial [ISRCTN45828668]	[108]
GS inhibition	actinomycin D or 5-azacytidine	D54MG human GBM cell line; TE671 human medulloblastoma cell line	decreased proliferation	[72]
GS induction	GS overexpression	C6 rat glioma cell line	decreased proliferation and migration	[120]
GLUD inhibition	GLUD1/2 shRNA	mouse intracranial xenograft (mouse glioma progenitor cells *IDH1^R132H^*)	decreased tumor volume	[121]
GLUD2 induction	GLUD2 overexpression	human T98G GBM cell line	decreased proliferation and migration	[122]
reversing lactate effects	LDHA siRNA	U87MG and U251MG human GBM cell lines	decreased proliferation; increased sensitivity to TMZ	[132]
	NHI-1, NHI-2	U87MG, U343MG, T98G, ANGM-CSS human GBM cell lines and U87MG-derived GSCs	decreased proliferation and sphere formation; increased GSCs differentiation toward neuronal/glial phenotype	[134]
